# KiESEL – The Children’s Nutrition Survey to Record Food Consumption for the youngest in Germany

**DOI:** 10.1186/s40795-022-00527-6

**Published:** 2022-07-14

**Authors:** Nicole Nowak, Friederike Diouf, Nadine Golsong, Tobias Höpfner, Oliver Lindtner

**Affiliations:** German Federal Institute for Risk Assessment, Berlin, Germany

**Keywords:** Food consumption survey, Children, Food propensity questionnaire, Dietary record, Consumption data, Seldom eaten foods

## Abstract

**Background:**

With KiESEL, the Children’s Nutrition Survey to Record Food Consumption, the German Federal Institute for Risk Assessment (BfR) collected representative food consumption data for children aged six months up to five years. KiESEL was one of five modules of KiGGS Wave2 (German Health Interview and Examination Survey for Children and Adolescents) conducted by the Robert Koch Institute (RKI). The objective was to update the consumption data for children in Germany and to fill a data gap for the age group of five-year-old children. The study provides an up-to-date and comprehensive database that will be used for exposure assessment, as part of risk assessment of Germany’s youngest consumers.

**Methods:**

In the years 2014 to 2017, 1104 children from all over Germany participated in KiESEL. During home visits, survey staff conducted a questionnaire-based interview including a food propensity questionnaire (FPQ) on seldom eaten foods and questions concerning consumption outside home, dietary habits and diet during first year. The interviewer measured the children’s height and weight. Families and childcare workers filled out a food record, covering three consecutive days and one independent day. Data are based on the FPQ and present consumption frequencies. Depending on the question, socioeconomic status (SES) and migration background were considered.

**Results:**

1104 participants had an interview and filled out the questionnaire on usual food intake, seldom eaten foods and consumption away from home. They were included in sample1. 1008 of these participants additionally reported food consumption of at least three days (sample2). 91.2% of the children follow no special diet and 0.8% are vegetarians. 7% of the older children consuming soya-drink. For some foods differences in consumption across SES or migration status were noted. Children from families with higher SES consume more often soya-based substitute milk as families with lower SES (p < 0.00005).

**Conclusions:**

KiESEL gathered up-to-date consumption data for more than 1000 children living in Germany, aged six month up to including five years. The data will be used for risk assessments of the BfR and provided to national and international partners.

**Supplementary Information:**

The online version contains supplementary material available at 10.1186/s40795-022-00527-6.

## Background

The German Federal Institute for Risk Assessment (BfR) carries out scientific risk assessments of substances in food as part of consumer health protection. With the background of risk assessment, exposure estimates are based on content data of substances and consumption data of the population. Higher exposure in relation to body weight are often found in children compared to adults. Therefore, young children are a particularly vulnerable group in the population [1–3]. The last consumption survey with a focus on risk assessment was carried out nearly 20 years ago in 2001/2002 in the 6-month to 4-year age group with the VELS study (consumption study to determine the food intake of infants and young children to assess an acute toxicity risk from pesticide residues) [4]. 5-year-old children were not included in VELS. In 2008, the GRETA study (German Representative Study of Toddler Alimentation) of the Research Institute of Child Nutrition (FKE) collected representative consumption data for children aged 10–36 months [5]. These data can only be used for risk assessment to a limited extent, as the food was recorded in very broad categories and children older than 3 years were not considered. In order to describe current food consumption, to evaluate revised nutritional recommendations [6], to take into account steadily increasing food offers (e.g. superfoods) as well as changes in global nutritional habits [7] an update of the consumption data of young children aged between 6 months and inclusive 5 years in Germany is necessary.

The Children’s Nutrition Survey to Record Food Consumption (KiESEL) was created and carried out by BfR as one of five modules of KiGGS Wave 2 (German Health Interview and Examination Survey for Children and Adolescents) [8] conducted by the Robert Koch Institute (RKI). The KiGGS study is part of the health monitoring system at the RKI [9]. The cooperation between the BfR and the RKI makes it possible to combine the consumption data from KiESEL with more comprehensive data from KiGGS wave 2. The generated data set combines nutrition and health data and thus more extensive evaluations are possible.

The main objective of the KiESEL study was to obtain food consumption data for young children for the purpose of estimating exposure to substances in food for example contaminants, pesticides or microbial risks required for health assessment. In this manuscript we describe the study design in general and methods we used in KiESEL. The focus of this publication and the presented results are based on the Food Propensity Questionnaire (FPQ) and present consumption frequencies.

## Methods

### Study design and study population

KiESEL was conducted all over Germany from December 2014 to October 2017. In the first step, 167 sample points, representative cities and communities for the Federal Republic of Germany, were selected for the study population of KiGGS Wave 2. The target population and sampling for KiGGS Wave 2 is described in detail elsewhere [8]. Infants, toddlers and young children were selected randomly from the participants of the cross-sectional KIGGS Wave 2 sample for participation in the KiESEL study.

The study aim was to achieve at least 1000 participants. A case number of 167 children per year was aimed for so that approximately 83 participants each were planned for age and gender. The age of study participants at time of recruitment by RKI differs from the age at the time of investigation due to the elapsed time between both parts of the survey. Children who grew older than 5 years until the home-visit (*n* = 64) were not excluded. For this article the participants were age classified based on their age at time of the KiESEL interview.

Participation in the study was optional. An interview was conducted after information and written consent of the legal guardians of the participating children. The study is approved by the Federal Commissioner for Data Protection and Freedom of Information and by the ethics committee of the berlin medical association (Eth-28/13). KiESEL was also audited by „aproxima—Gesellschaft für Markt- und Sozialforschung Weimar mbh “ as part of an external quality management system.

### Study procedure

**Fig. 1:**
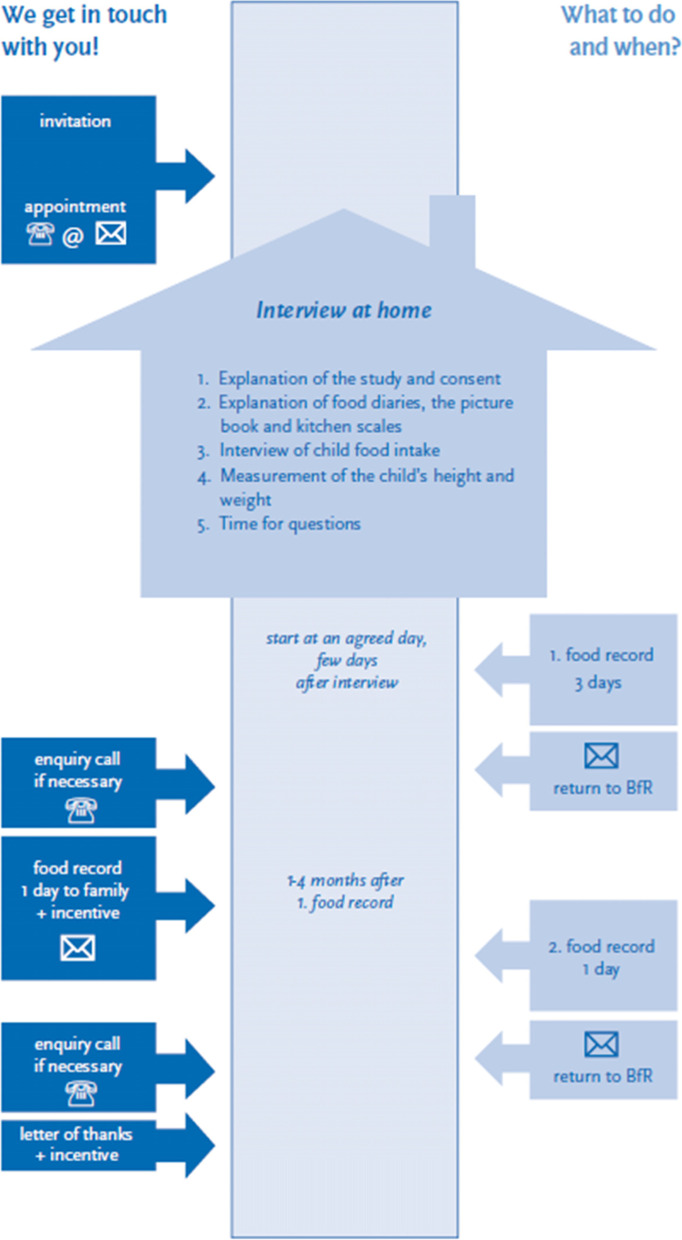
Study procedure KiESEL

KiESEL's methods are based on the study protocols of EsKiMo I (Eating Study as a KiGGS module) and the VELS study [4, 10, 11]. The detailed study procedure of each participant is shown in Fig. 1 [3]. After making an appointment by phone or email, trained nutritionists visited the participants at their home. According to the protocol, the visit was identical for each participant and took about 1 h (mean 65 min and standard deviation 13 min, based on 1079 questionnaires where duration was indicated). During this time, an interview was conducted, instruction was given on how to complete the two food records, kitchen scales were provided, the children were measured in height and weight and open questions were answered.

The route planning should achieve a nationwide representativeness in terms of both seasonality and regionality and followed the sample points of KiGGS [8]. During the field phase a total of 117 tours in 167 sample points around Germany were approached. The visits took place exclusively at the participants' homes. The opportunity to conduct the interview in the KiESEL bus was offered to all participants, but was chosen only once.

### General questionnaire and FPQ

The interview concerning the child’s food intake was carried out by means of a questionnaire. The questionnaire included general information (e.g. gender and age), the child’s current dietary habits, information on its diet during the first year, the out-of-home-consumption, a FPQ on seldom eaten foods and further questions for self-completion on the parents’ attitude towards nutrition. More details to the topics of the questionnaire are described here [3]. We developed this special FPQ due to our experience in risk assessment. Therefore, we asked about the frequency of consumption of seldom eaten foods in the last 12 month, such as offals from various animals or foods due to risk assessment like various tea and herbal infusions. These consumption data are of particular interest for the risk assessment, e.g. to know the possibility of high concentrations for some contaminants (e.g. pyrrolizidine alkaloids in tea [12]. Its intention is to identify non-consumers of certain foods and to reflect the eating frequency of seldom eaten foods that are often underrepresented in other food consumption methods like dietary records or 24 h-recalls [13]. The FPQ contains 93 different food items. All food items are shown in a supplementary file. The question in the FPQ always started with “How often has your child consumed the following foods since the introduction of complementary foods (for children ≤ 18 months) or in the last 12 months (for children > 18 months)?”. For a better overview, the answering categories were simplified for the analysis (Table 1).

**Table 1:** Frequency categories as asked in the questionnaire and as aggregated for analysis

Category in the questionnaire	Category in the analysis
Never	never consumed
Less than once a month	rarely consumed
1–3 times a month	
once a week	frequently consumed
2–3 times a week	
4–5 times a week	
6–7 times a week	
no comment	no comment

The analysis were performed looking at the EFSA age groups: infants (< 12 month), toddlers (12 month to < 36 month) and children (> 36 month) [13]. If analyses were performed regarding influence of socio-economic status (SES) or migration background, these variables were taken from the KiGGS dataset. Participants were classified as “with migration background” if one or both of the parents was not born in Germany. The SES was derived from the factors of education, employment status and parental income. The participants were categorized into three SES groups (low, middle, high) [14].

In general, this questionnaire provides additional information to the food records. Filling it out in paper form during the home visit enabled the parents to get their questions answered directly by the interviewer. Even though all interviewers where nutritionists there was an interviewer's guide to ensure a standardized interview procedure, which clarified the background to the questions and questions of understanding regarding the contents of the questionnaire in general. The last part of the questionnaire included questions about the general choice of food (e.g. organic or conventional) and its preparation. Self-completion was chosen only for this part to avoid a bias in the answers due to social desirability and the feeling of possible judgement by the interviewer. After the home visit the data were entered into the web-based application LimeSurvey [15].

Beside to the general questionnaire and the FPQ, the food intake of the children was prospectively documented by the families and, where applicable by child day care facilities in form of a weighing and estimation dietary record for three consecutive days and an unrelated fourth day. The food record for day care workers was designed in a less detailed way (see section “Pretest”). Participants without a dietary record were not excluded from the survey. Therefore we differ between sample1 (questionnaire) and sample2 (questionnaire and dietary record for at least 3 days). Methodology and results of the dietary record will be published in a separate article.

#### Anthropometry

To obtain reliable values for the child's weight and height, standardized measurements were taken [16]. The height and weight of infants was measured while the child was lying down using a portable measuring board and the body weight was measured using a portable and calibrated infant scale. For children who could stand securely, the height was measured while standing with a portable stadiometer and the body weight with a portable and calibrated scale. The participants were weighed once without clothes, only with underwear on. In case clothes could not be taken off or for the smaller ones empty nappies should stay on, a certain amount of the weight was subtracted.

If the participant refused weighing or measuring the possibility existed to use the information from the medical examination booklet all children in Germany own and where all regular examination results can be found. This occurred 35 times for body weight and 38 times for height. In some other rare cases (around 20 participants) self-declarations from the parents were accepted.

### Pretest

The aim of the pretest was to test the developed survey instruments as well as the information materials for comprehensibility and feasibility [17]. This included contact with the participants, the quality and handling of the survey instruments, the comprehensibility and duration of the questionnaire as well as the communication with care institutions (child day care centre, kindergarten etc.). The participants wrote down their experiences and comments in two satisfaction questionnaires, which were then evaluated by the KiESEL team. In this context the time schedule of the study and the general effort of the participants were tested. A total of 40 families from Berlin and Brandenburg took part in the pretest, 4 age groups were formed to determine the age-specific dietary requirements. The study instruments were refined based on the results of the pretest (data not shown). In general, to facilitate all processes, an additional graphic explanation was developed to enable the participants to fill out the dietary records more easily. For the use of the picture book, graphic instructions were embedded and a keyword register for substitute foods was added. As another result, the dietary record for the child day care was simplified and only the most necessary columns were kept, e.g. time and place of consumption, product description with brand and specific product name, organic/conventional and the amount eaten. There was still enough space to note recipes in case the child day care workers prepared all meals by themselves or to mention the caterer. Additionally a specific flyer with all the necessary information for the child day care centres was created.

### Dietary supplement database

In order to satisfy the requirements of the risk assessment, a dietary supplement database was created, implying all data from the food records and the questionnaire referring to dietary supplements. The database is divided into groups like single and dual nutrient preparations with specification, multivitamins, minerals, multi-nutrients and multi-fatty acid preparations, probiotics and others without specification. The nutrient composition of all specified dietary supplements was checked with the available product information from the photographed packaging, the Internet, other available databases and, if available, manufacturer information. More detail can be found in a separated paper about dietary supplements in KiESEL [18].

### Representative Sample Weighting

In order to ensure that estimates based on the study population are representative for Germany in the respective age group a weighting variable was prepared. This includes for sample 1 the variables age, sex, region, regional structure (size of a municipality), distribution of weekdays and parental education. The sample weighting takes into account the different participation probabilities within the sample design. It corrects deviations of the design-weighted net sample from the German population using population statistics from 2014/ 2015 and the distribution of education according to the CASMIN classification (Comparative Analysis of Social Mobility in Industrial Nations) from the 2013 micro census. Individual sampling weights were made available by courtesy of the KiGGS team of RKI.

### Statistical Procedures

Characterization of the study population was based on sex and age. For this purpose the participants were classified according to their age in infants (< 12 months), toddlers (12 – 35 months) and children (> 35 months). Information on frequencies include percentages and numbers, if appropriate. Kruskal–Wallis H test was used to assess differences for consumption frequencies between groups of socio-economic status (SES), with SES modelled as grouping variable (low SES, middle SES and higher SES). To ascertain which pairs of groups of SES differ from each other additional post hoc analyses were performed (Bonferroni correction). The Mann–Whitney U test was used to determine differences for consumption frequencies between participants with migration background and participants without migration background. In case of missing values or abstentions on SES, migration status or consumption frequencies participants were excluded from the analyses described above. A P value less than 0.05 was considered to indicate a statistically significant difference. Statistical calculations were performed by using IBM SPSS Statistics (Version 26) and Microsoft Excel 2016. All presented results are based on weighted data (otherwise mentioned in the explication).

## Results

### Sample description

#### Participants and response

**Fig. 2:**
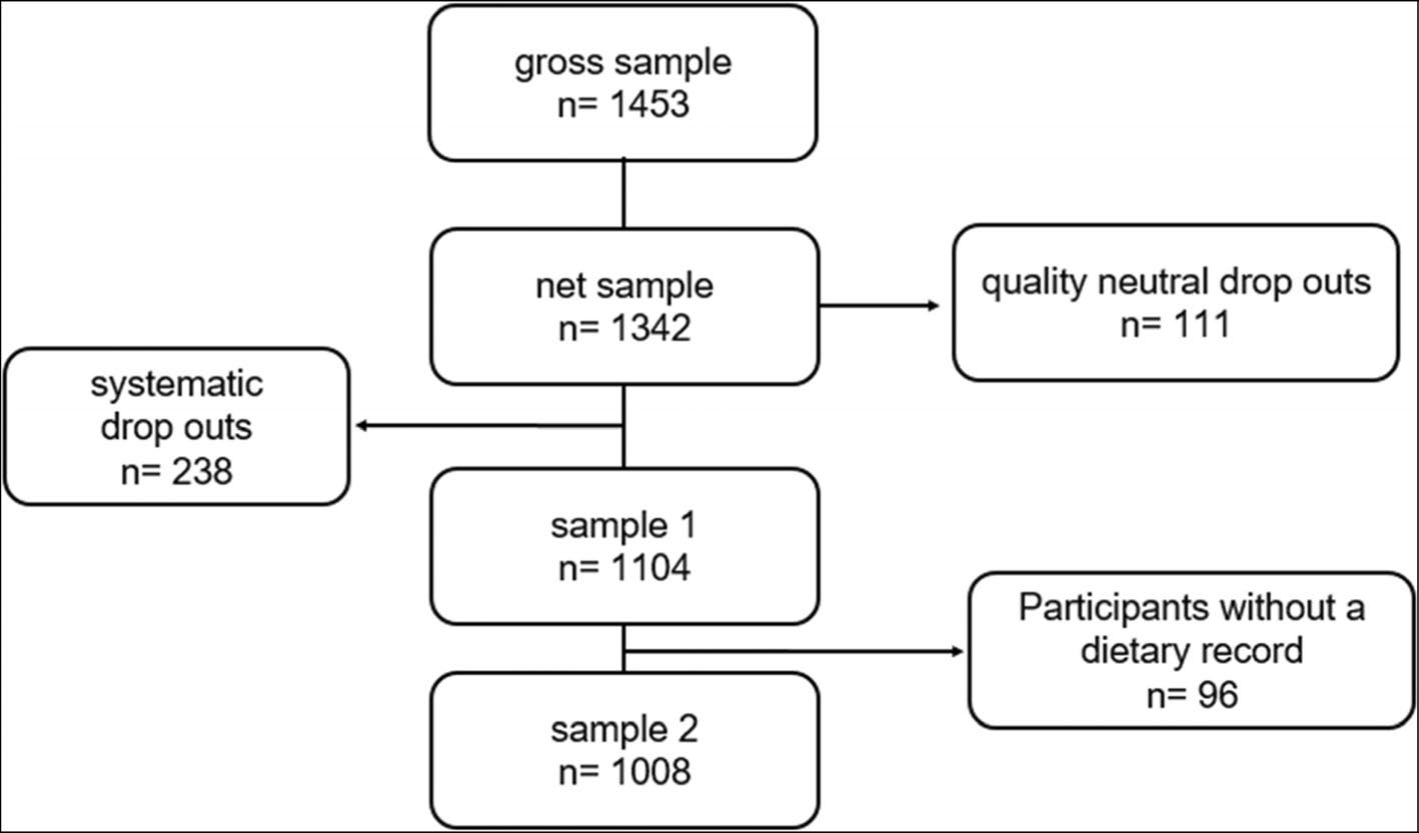
Overview of participants and non-participants

The KiESEL study comprises two study samples. In sample1 there are all 1104 participants that had an interview, filled out the general questionnaire, the FPQ and anthropometric data. In sample2, a subsample of sample1, there are those 1008 participants that reported at least 3 days of their food consumption, additionally to the interview. Response rate for sample1 is 82% and for sample2 it is 75%. More details of participants and drop outs are summarized in Fig. 2.

#### Age

Sample1 consists of 560 boys (respective to 565 boys using sample weighting) and 544 girls (respective to 539 girls using sample weighting) and sample2 consists of 510 boys (respective to 515 boys using sample weighting) and 498 girls (respective to 492 girls using sample weighting). In both samples, the number of children for every age group aimed for was achieved or surpassed except for the under-one-year-olds and the two- and three-year-old girls. Equality in gender distribution was nearly achieved as well. For the one year-olds, the under one-year olds and the more than five-year-olds there were always slightly more girls than boys and for the two-, three-, four- and five-year-olds there were slightly more boys than girls (Table 2).

**Table 2:** Age distribution in KiESEL – sample1 and sample2

	**Sample1**			Sample2**		
	Male	Female	Total	Male	Female	Total
0 y	59	64	123	56	62	118
	42*	45*	88*	37*	42*	79*
1 y	92	116	208	80	110	190
	99*	86*	186*	90*	85*	175*
2 y	99	76	175	95	69	164
	93*	87*	181*	86*	80*	166*
3 y	89	77	166	79	68	147
	91*	96*	187*	88*	86*	174*
4 y	97	90	187	85	78	163
	103*	88*	191*	86*	76*	162*
5 y	95	86	181	87	77	164
	103*	103*	206*	98*	91*	189*
> 5 y	29	35	64	28	34	62
	33*	33*	66*	30*	32*	62*
Total	560	544	1104	510	498	1008
	565*	539*	1104*	515*	492*	1007***

^*^weighted numbers

^**^sample2 is a sub-sample of sample1

^***^difference in total numbers are caused by rounded weighted numbers

#### Eating habits

As to know more about the eating habits the study population can be characterized by the special diets participants were used to e.g. vegan, halal, lactose-free diet. The majority follows no special diet (91.2%). A vegan diet was chosen by 0.4% of the participants whereas 0.4% more crossed this answer but showed no consistency in the food protocol. This additional 0.4% was not considered as vegan. 0.8% of the participants used a vegetarian diet. 1.8% of the participants followed a lactose-free and 0.4% a gluten-free diet. 0.5% of the children were used to a diet poor in fructose, whereas 0.8% were used to a diet poor in milk products and 1.1% tried to avoid foods containing nuts, eggs, wheat, fish or stone fruits due to allergy. 2.0% of the participants only ate halal food. 1.7% didn´t answer this question and the answer of 0.1% was “other diet”. In sample2 these findings are very similar.

#### Socio-economic status and education

Looking at the socio-economic status 61% of the participants belong to the category middle, 16% to the category low and 23% to the category high socio-economic status. Percentages are similar regarding the parents’ education level (51%, 16% and 33%, respectively). Sample2 shows similar findings with slightly less participants with lower socio-economic status and lower educational level.

#### Out-of home consumption and day care

KiESEL is one of the first studies taking into account not only the food eaten at home but as well the food eaten outside home (e.g. restaurants) and in child day care. The importance of covering the child day care can be seen in the number of children being cared for in day care facilities: this are more than half of the KiESEL participants aged 1 and 2 years (58,2%) and nearly all of the KiESEL participants aged over 3 years (97,5%). Most children under 1 year of age are cared for at home (93.3%).

### Results of the FPQ on seldom eaten foods

Here, only selected results are presented in detail and results for all other food items of the FPQ was made available in a supplementary file.

- Complementary food

**Fig. 3:**
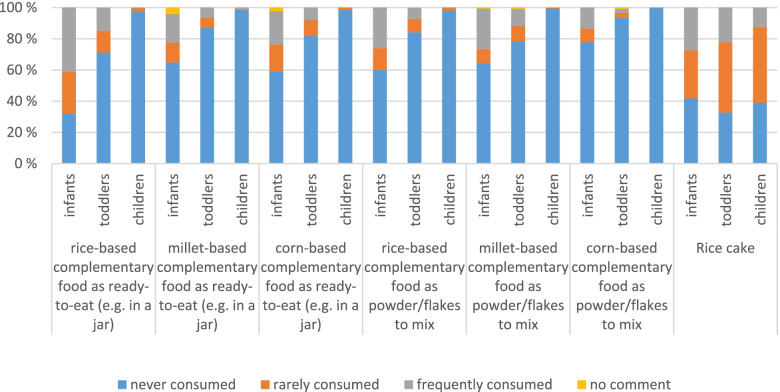
Frequency of complementary food consumption, differentiated for the different kind of cereal bases

All complementary foods are mostly consumed by infants (22–68% consumers), a little by toddlers (5–29% consumers) and rarely by children (0–3% consumers) (Fig. 3).

Even though the cereals presented in Fig. 3 are considered to be rarely consumed in Germany it should be noted that for all of the complementary foods more than 6% (up to 37% for rice-based complementary food as ready-to-eat or up to 18% for corn-based complementary food as ready-to-eat) of the infants consuming it least once a week. A supplementary table “Consumption frequency of complementary food, differentiated for the different kind of cereal bases” shows the detailed data. See supplementary table_1.xlsx. Most infant consumers can be found for rice-based complementary food as ready-to-eat: 41% consume it frequently (37% even once a week) and 27% consume it rarely whereas 15% of the toddlers are frequent consumers of rice-based complementary food as ready-to-eat and 14% consume it rarely. Millet- and corn- based complementary food as ready-to-eat is not consumed that frequently: 19% of the infants are frequent consumers of millet-based complementary food (and 13% are rare consumers) and only 6% of the toddlers consume it frequently whilst 7% consume it rarely. Complementary food as powder/flakes is used a little less frequently for nearly all kind of cereals asked for. Rice-based complementary powder/flakes are consumed frequently by 26% of the infants and 7% of the toddlers, millet-based complementary powder/flakes are frequently consumed by 26% of the infants and 11% of the toddlers and corn-based complementary powder/flakes are frequently consumed by 14% of the infants and by only 3% of the toddlers.

The most consumers of rice cake can be found in between the toddlers, followed by children and infants. Nevertheless, if they consume it, infants consume it more frequently. From the 58% infant consumers: 27% consume it frequently and 31% rarely. Looking at the toddlers: 22% of them consume it frequently and 45% rarely. As for the children: 12% of them consume it frequently and 49% rarely.

b) Dairy substitutes (soya drink, rice drink, oat drink) and other soya-based products

**Fig. 4:**
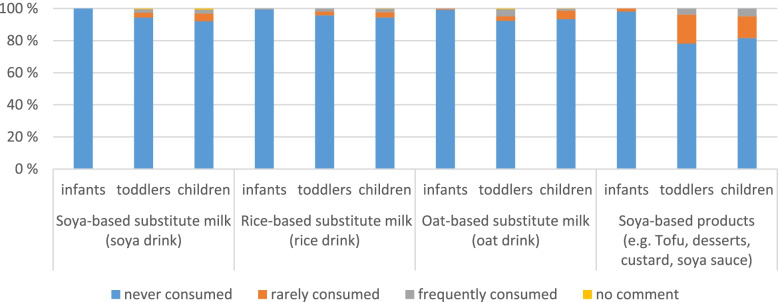
Frequency of consumption for different dairy substitute drinks and other soya based products, differentiated for the three age groups

Study participants do not seem to consume dairy substitute drinks very frequently. The maximum are 7% of the children consuming soya-drink and 7% of the toddlers consuming oat-drink. For other soya-based products (e.g. tofu, desserts, custard, soya sauce) there are 22% of the toddlers and 18% of the children consuming them (Fig. 4).

Oat drink seems to be the most common consumed substitute drink with more than 4% frequent consumers (mostly once a week) and 3% rare consumers for the toddlers and more than 1% frequent consumers and 5% rare consumers for the children. Soya based substitute drink follows with 5% rare consumers for the children and 3% for the toddlers and even nearly 3% frequent consumers for the children. Other soya-based products e.g. tofu on the other hand are consumed more often, at least by toddlers and children.

As the use of dairy substitutes may be influenced by socio-economic status (SES), we had a closer look on this influence for each substitute. It is visible, that children from families with higher SES consume more often soya-based substitute milk as families with lower (*p* < 0.00005) and middle SES (*p* < 0.015). We saw no significant influence by SES for consumption of rice drinks. The consumption of oat-drink was higher from children from families with middle (*p* < 0.048) or higher SES (*p* < 0.016) than by families with lower SES. For soya-based products, the range goes from 7% consumers for families with lower SES to 30% consumers for families with higher SES. It can be seen as well that the higher the parents’ education level, the higher the consumption of soya-based substitutes (lower SES to middle SES *p* < 0.006, middle SES to higher SES *p* < 0.0005).

c) Special meat and sausage products (game, sheep/lamb, ostrich, rabbit, horse)

**Fig. 5:**
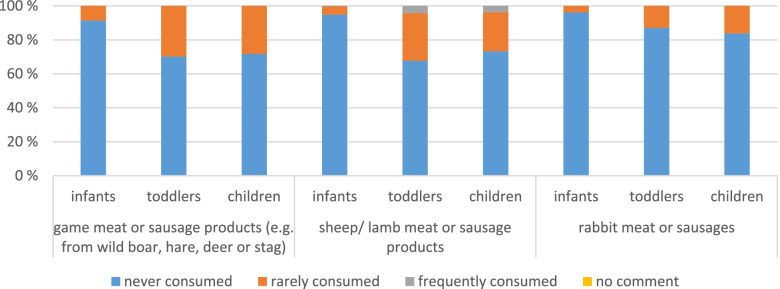
Consumption frequency of the different types of special meat and sausage products, differentiated for the three age groups

Consumption differences due to age and migration background are presented here. Looking at the age differences first in Fig. 5. Infants seem no big consumers of these types of meat (from 0% for ostrich and horse to 9% for game meat). Toddlers and children seem to consume game meat more often but most of the time rarely. There are 4% toddlers and children that consume sheep/lamb meat frequently. They eat it mostly once a week and some consumers as well 2–3 times a week. For all the other meat and sausage products, there are less consumers, and it is consumed mostly less than once a month. There are around 30% of toddlers and children consuming game meat. 27% of the children and 32% of the toddlers consume lamb/sheep meat. 13% of the toddlers and 16% of the children consume rabbit meat. Whereas horse meat and ostrich meat are nearly not consumed (and therefore not displayed here).

As the migration background may influence the type of meat consumed, analyses were performed taking into account the migration background. It can be seen that only for game meat and sheep/lamb meat there is a visible difference in consumption frequency. Even though game meat is consumed most often rarely, if it is consumed, it is more often consumed by participants without migration background (31% consumers vs. 17% consumers with migration background (*p* < 0.0005)). Whereas for lamb/sheep meat the relation is inverse (*p* < 0.0005). 45% of participants with migration background consume lamb/sheep meat rarely or frequently. Only 20% of participants without migration background consume this meat. There are 6% of the participants with migration background that consume it even once a week and 1% that consume it 4–5 times a week. Ostrich, horse or rabbit meat are consumed rarely and there are no differences regarding the migration background.

d) Offal

 

**Fig. 6:**
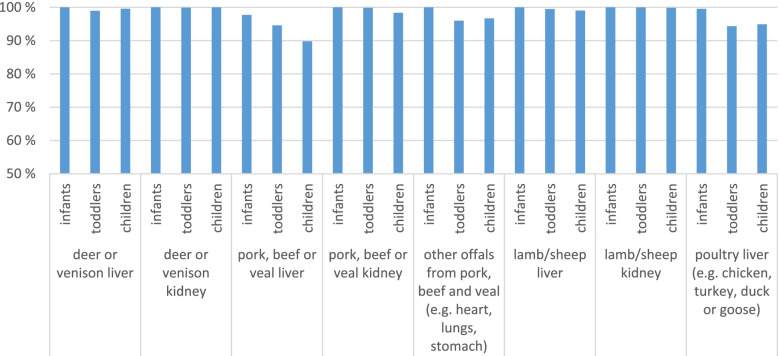
Non-consumers of offal from different animals, differentiated for the three age groups

Offal is known as a very seldom eaten food but often contains high levels of several contaminants and hence can be relevant for risk assessment. Results from KiESEL confirm that infants, toddlers or children seldom eat offal (Fig. 6). Only liver from pork, beef or veal and poultry liver seem to be eaten. If it is eaten, then nearly always less than once a month and more often by toddlers and children. Only 2% of the infants eat pork, beef or veal liver and do this less than once a month. Some other offal from pork, beef and veal (e.g. heart, lungs and stomach) are eaten by 4% of the toddlers and 3% children but mostly less than once a month.

e) Fish and other marine animals

 

**Fig. 7:**
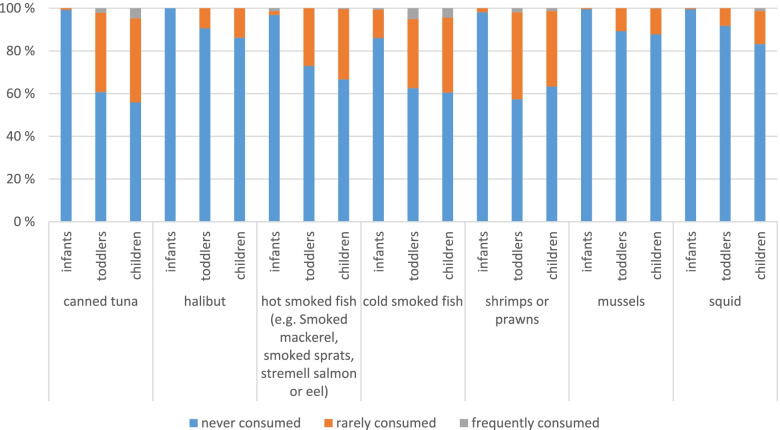
Consumption frequency of fish and marine animals differentiated for the three age groups

In the questionnaire, participants were asked about their consumption of 14 different fish and marine animal species. The questions with more than 10% consumers are presented in Fig. 7. Infants do not seem to eat the fish species asked for very often. Most likely, they eat cold smoked fish (14% consumers, mostly less than once a month). For all the other chosen fish species there are 0–3% infant consumers only. For toddlers and children most of the consumers can be found for canned tuna and shrimps/prawns. There are 39–44% consumers of canned tuna and 2–5% of them are frequent consumers (toddlers and children, respectively). For shrimps and prawns we found similar results even though toddlers seem to eat them slightly more frequently than children. However, there are a little less consumers for shrimps and prawns than for canned tuna. Toddlers and children as well seem to like cold smoked fish (37–40% consumers for toddlers and children, respectively). Even though most of them consume it less than once a month, there are around 5% toddlers who consume it once a week and 1% of the children even 2–3 times a week. Hot smoked fish, e.g. stremel salmon or smoked mackerel, follows in the list of the most popular fishes with 27–33% consumers (toddlers and children, respectively) but it is mostly consumed less than once a month. Mussels, squid and halibut show 89–17% consumers as well.

f) Tea and tea products

 

**Fig. 8:**
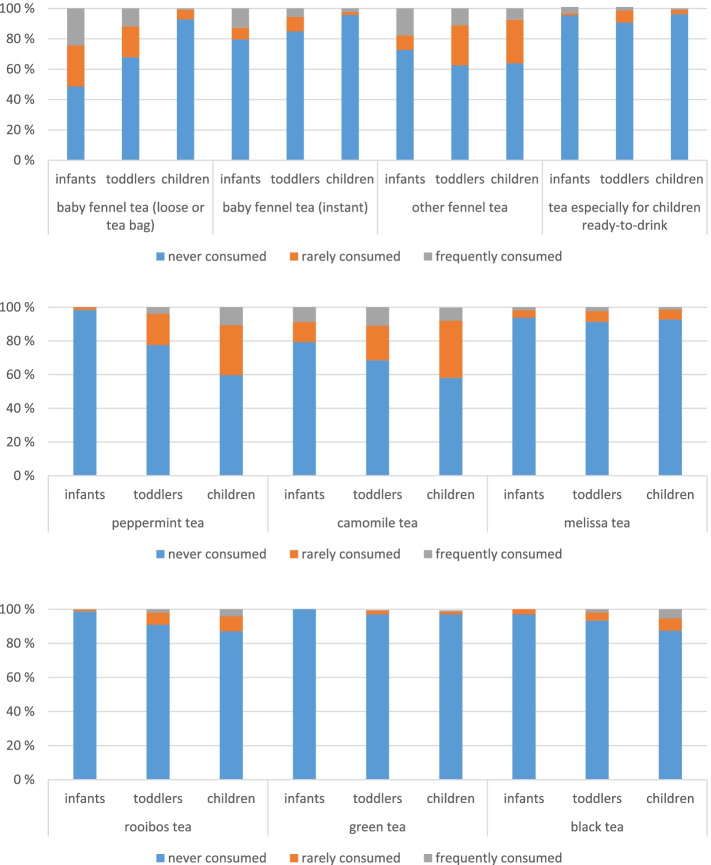
Consumption frequency of tea and herbal infusions, differentiated for the three age groups

Tea is presented in three groups which are all presented in Fig. 8: 1. Three different types of fennel tea and ready-to-drink tea especially for children, 2. Other herbal teas, 3. Rooibos tea, green tea and black tea.

1. Baby fennel tea (loose or tea bag), baby fennel tea (instant), other fennel tea and ready-to-drink tea

 

Infants and toddlers mostly consume baby fennel tea (instant and ready-to-drink). Toddlers and children mostly consume other fennel tea. One quarter of the infants are frequent consumers of baby fennel tea (loose or tea bag). 8% of the infants drink it even nearly every day. 13% of the infants are frequent consumers of instant baby fennel tea as well. Most of them consume it 2–3 times a week but 5% of the infants consume it nearly every day. This applies to 2% of the toddlers and 1% of the children as well. Nearly 40% of the toddlers and children drink other fennel tea. For the infants there are 27% other fennel tea consumers all together and 18% of them frequent consumers (3% consume it nearly every day). 11% of the toddlers and 8% of the children are frequent consumers of other fennel tea and they consume it most often several times a week. Ready-to-drink tea especially for children is not consumed very often. There are no more than 10% consumers (toddlers) and it is mostly consumed rarely, nevertheless there are 3% of the infants who consume it nearly every day.

We wondered whether the socio-economic status of the families had an influence on the choice of fennel tea. Statistical significant differences existed only for baby fennel tea (instant). Families with middle and higher SES (7% and 4% consumers, respectively) consume it, whereas 25% of the families with lower SES consume it. Differences in consumption frequencies for fennel tea (instant) are significant between families with lower and higher SES (*p* < 0.0005) and between families with lower and middle SES (*p* < 0.0005). Most of them with lower SES drink it frequently (16%), 5% drink it nearly every day.

2. Herbal teas (peppermint, camomile and melissa)

Infants consume peppermint tea or melissa tea rarely. For peppermint tea there are only 2% consumers (mostly less than once a month) and for melissa tea there are 4% less frequent and 2% frequent consumers. These melissa tea numbers are similar for toddlers and children. Peppermint tea, on the other hand, is more popular with toddlers and children (22% and 40% consumers, respectively). There are even 11% of the children that consume it frequently, most of them once a week. 21% of the infants, 31% of the toddlers and 42% of the children consume camomile tea, most of them rarely. Nevertheless, there are between 8–11% who drink it frequently, the little ones mostly 2–3 times a week, the older ones mostly once a week.

Looking at the social-economic influence we only see significant values in camomile tea: The most consumers of camomile tea are from families with lower SES (46% consumers) and 20% of them consume it frequently. The differences in consumption frequencies were shown between higher and middle SES to lower SES (*p* < 0.005 and *p* < 0.002, respectively).

3. Rooibos tea, green tea and black tea

KiESEL-participants do not consume rooibos tea, green tea or black tea very often. Green tea is consumed the less, by only 3% of the toddlers and children. 3%, 7%, 13% of the infants, toddlers and children, respectively, consume black tea. It is consumed most often less than once a month or 1–3 times a month, but there are 2% of the children who drink it nearly every day. The consumption of rooibos tea is similar to the consumption of black tea. If it is consumed frequently, then mostly once a week.

Concerning the socio economic background of the participants, black tea was consumed more by families with lower SES in comparison with middle SES (*p* < 0.002) and also in comparison with higher SES (*p* < 0.0005). 18% of the participants with lower SES are consumers of black tea and 6% of them consume it even frequently (1% drink it nearly every day). Whereas families with higher SES show 4% consumers and families with middle SES show 9% consumers.

For some of the tea species migration status might play a role as well. For other fennel tea (*p* < 0.0005) the participants without migration background show a significant higher consumption. For baby fennel tea (instant), there are no significant differences for participants with or without migration background. Peppermint tea (*p* < 0.022) and melissa tea (*p* < 0.001) were consumed more by participants without migration status. Whereas camomile tea shows higher consumption for participants with migration status (*p* < 0.0005). The consumption of Rooibos tea shows no significant differences between both groups. Participants with migration status also show higher percentages for consumption of green tea (*p* < 0.049) and black tea (*p* < 0.0005). Especially the percentages of frequent consumers are higher for participants with migration status (1.6% for green tea and 10.2% for black tea). For black tea, there are only around 4% consumers of the participants without migration status whereas around 24% of the participants with migration status consume black tea (3.8% of them consume black tea nearly every day).

As for regional differences (categorization of regions in KiESEL see ST11 in the supplementary file): In the north-western part exists the highest percentages of black tea consumers. This difference is significant for all regions (16%, eastern part *p* < 0.0015; North Rhine-Westphalia *p* < 0.021; Bavaria/Baden-Württemberg *p* < 0.038) except for the middle of Germany (11%). The participants in north-west also drink it most frequently: 5% drink it 2–3-times a week, 2% drink it nearly every day.

g) Diverse foods and food groups

In these questions we asked for many different food items. We choose the following to have a closer look at. The detailed data of these diverse foods are shown in the supplementary file.

1. Sweeteners, products with stevia, calorie-reduced drinks

 

Sweeteners are only consumed by around 5% of the toddlers and children (not at all by infants) and mostly less than once a month. Only 0.4% of the infants consume food sweetened with stevia (less than once a month); 4% of the toddlers and 6% of the children consume it rarely and 2% consume it frequently (mostly once a week or 2–3 times a week). Calorie-reduced soft drinks were nearly not consumed by infants whereas around 8% of the toddlers and 21% of the children are calorie-reduced soft-drink consumers. 6% of the children consume it even frequently (around 1% nearly every day).

Focused on all consumers there are no differences in the frequency of consumption of calorie-reduced soft drinks for all three socio-economic statuses. Nevertheless, it shows that the percentages of frequent consumers are higher for participants from families with lower SES (11% for families with lower SES, 4% for families with middle SES and 1% for families with higher SES). Nearly all daily calorie-reduced soft drink consumers are from families with lower SES. Participants from families with middle or higher SES consume soft drinks with sweeteners mostly less than once a month or 1–3 times a month (Table 3).

**Table 3:** Influence of SES for consumption frequency of calorie-reduced soft drinks

	**Socio-economic status (SES)**			**Total**
	**Low**	**Middle**	**High**	
Calorie-reduced softdrinks				
Never	83.80%	85.20%	83.80%	84.70%
< once a month	1.70%	6.70%	12.60%	7.30%
1–3 times a month	3.50%	3.90%	1.60%	3.30%
Once a week	3.50%	2.80%	0.40%	2.40%
2–3 times a week	2.90%	0.90%	0.40%	1.10%
4–5 times a week	0.00%	0.30%	0.00%	0.20%
6–7 times a week	4.60%	0.10%	0.00%	0.80%
No comment	0.00%	0.00%	1.20%	0.30%

Differences in frequently consumption of calorie-reduced soft drinks were significant for participants with lower SES and middle SES (*p* < 0.003). Due to the low number of cases, frequently consumers of calorie-reduced soft drinks with higher SES were not included in these analysis.

Results for energy drink consumption surprised as 1.6% of the children (> 3 years old) already consumed it. All of them consumed it less than once a month. Two thirds of those who already consumed it are from families with lower SES.

2. Rocket salad and fresh spinach

 

Infants quasi do not eat rocket salad and fresh spinach (as salad). There are only 3% rocket salad consumers and if they eat it, then most often less than once a month. Around one third of the toddlers and children eat rocket salad (mostly less than once a month). Around 3% eat it once a week. Fresh spinach is eaten even less. Only 12% of the toddlers and children consume it (mostly less than once a month).

3. Dark chocolate

 

Infants rarely eat dark chocolate. Only 12% consume it and they consume it less than once a month. There are more than 50% consumers in both age groups and 7% of the toddlers and 8% of the children eat dark chocolate even frequently (mostly once a week).

Tables of analysis for all the other food items that are not described in this manuscript can be found in the supplementary file.

## Discussion

The aim of the study was to generate an up-to date and representative database for children in Germany from 6 month up to inclusive 5 years old. This database will be used for exposure assessment, so the survey methods were adapted to fulfil the respective requirements.

### Sample description

The efforts made to get target numbers of participants were successful. Especially contacting the future participants by phone was much more effective than sending invitation letters. This direct contact results in the high response rate of 82%, which has a positive effect on representativeness. The age distribution in general as well as separately analysed for gender resulted in numbers as aimed for with only little deviations and also in a good adaptation to the German population. As visible in the results, there were less under-one-year-olds, more one-year-olds and some more over five-year-olds. These results were expected and are due to the time shift between recruitment and investigation. People with higher education and socio-economic status are found more often to be interested in nutrition and health issues and therefore are often more willing to take part in nutrition studies [13]. This can also be seen in the results of the KiESEL study as well where 83% of the study population show middle or higher (parental) education and socio-economic status. In comparison with national statistics of being in a child day care facility, the numbers from KiESEL are comparable (national statistics to KiESEL 0 < 3 year old children: 33% to 48% in child day care respectively > 3 year old children: 94% to 98% in child day care) [19]. With the given sample size of 1104 subjects, an optimal fit of all variables to the population distribution cannot be achieved. The sample contains only 28 foreigners. Therefore, no variables on the status of foreigners or on migration background were taken into account in the weighting. In general, children from large cities are a little bit underrepresented in KiESEL, even after weighting (data from KiGGS wave 2 not shown). Nevertheless, as one of the aims was representativeness concerning the region, the weighted results show also a very similar distribution among the federal states to the data of the Federal Statistical Office.

#### Eating habits

The majority of the participating children follows no special diet. Special diets as vegan or vegetarian diet are getting more in focus of public and scientific attention. Representative data for Germany of vegetarian fed children from 6 month up to inclusive 5 years were not known so far [20]. In KiESEL, 0.8% follows a vegetarian and 0.4% a vegan diet. Because of less numbers, it wasn´t reasonable to stratify for sex or SES. For older children in Germany, the prevalence is higher. EsKiMo II observed a vegetarian (incl. vegan) diet by 1.4% of 6- to 11-year-olds and 5.0% of 12- to 17-year-olds. The children and adolescents following a vegetarian diet tends to increase with increasing SES, but the results are not significantly [21]. The German Nutrition Society don´t recommend a vegan diet throughout childhood and adolescence [22], which might be one explanation for the lower percentages in the younger population.

#### Food record in day care facility

As more than half of the KiESEL participants aged 1 and 2 years and nearly all of the KiESEL participants aged 3–5 years used to go to a day care facility, it was very important to have the food records for these consumption times as well. KiESEL is one of the first studies to take into account the out of home consumption, which provides important information for a better understanding of children consumption structure. In the Dutch National Food Consumption Survey the parents or caregivers filled out a food diary the day before the dietary recall took plays [23]. As the numbers show, depending of the age, for 50 – 100% of the study population their consumption was only completely recorded using the two forms of the food protocols. From this results it becomes obvious that it is essential to use reliable methods.

### Food Propensity Questionnaire

The results of the questionnaire on seldom eaten foods is intended to be used in future exposure assessment by analyzing food consumption from the dietary records in KiESEL and additionally use the information from the FPQ. A combination of these dietary assessment methods is recommended to generate high quality data [24]. Usually a Food Frequency Questionnaire (FFQ) asks for food groups with a high aggregation level instead of single food items [25]. Due to the specific food items in the KiESEL-FPQ, there are no data available to compare with. Comparable results from EsKiMo II using the same FPQ as in KiESEL have not yet been published. The previous study, VELS used a 3-days-weighing record twice [4]. The GRETA study used an estimation food record for 7 days [5]. The consumption in DONALD were recorded with a 3-days-weighing record [26]. The German Environmental Survey for Children and Adolescents (GerES V) – the environmental module of KiGGS Wave 2 asked for dietary habits in the last 4 weeks [27]. Based on this, we would like to discuss the results in the view of the importance in risk assessment of several substances.

According to the results presented above, *complementary food* based on rice has the most infant consumers of all complementary foods asked for and should be carefully investigated in risk assessment of arsenic compounds and considered in risk assessments of possible contamination of complementary food as powder/flakes with mycotoxins [28, 29]. Current data from the BfR-MEAL-Study show higher content of inorganic arsenic in rice cakes/wafers than in white or brown cooked rice [30]. Therefore, also the fact that *rice cakes* seem to be quite popular in all age groups of the KiESEL-study is of relevance from risk assessment perspective.

*Dairy substitute drinks like soya, rice or oat drinks* do not seem to play an important role in the eating pattern of younger children. *Other soya-based products* e.g. tofu on the other hand seem to be slightly more often consumed as different dairy substitute drinks. It can be seen that if they are consumed than mostly by families with middle or higher socio-economic background. As most often SES goes together with education level, it could be seen that the higher the parents’ education level the more frequently these dairy substitute are consumed. Consumption of dairy substitutes might be not only a question of money but as well a question of awareness. We found more dairy substitute consumers within the families with middle and higher parents’ educational level and that they consume it more frequently. Similar results were found in literature for vegetarian diet in general [31].

It can be seen that *special meats* are consumed rarely and that especially ostrich and horse meat are (nearly) not consumed. For other meat, age and migration background seem to be influencing factors on the meat choice. Especially game meat and lamb/sheep meat consumption are influenced by (lacking) migration background. Extreme levels of lead have been reported in game meat, possibly due to lead from ammunition. However, infrequent consumption of game meat will not lead to health problems in the general population [32]. Infants, toddlers and children also show no big preference for *offal*. It is consumed extremely rare and only those of pork, beef, veal or poultry are consumed in KiESEL. The maximum permitted levels for dioxins and dioxin-like compounds in livers from beef, pork, sheep and poultry have been increased in 2013. The BfR recommends that the consumption of especially sheep liver should be avoided because of the possibly resulting higher intake of dioxins and dioxin-like compounds [33]. For future consumption surveys, a particular focus should be placed on specific consumer groups who generally consume more e.g. special meat and offal.

The selection of *fish species* we asked for were risk orientated and not the common fish preferred as dish. Therefore, the most fish species in the FPQ known to have high concentrations of heavy metals are rarely consumed by children in KiESEL. Whether they can significantly contribute to exposure needs to be checked based on dietary protocols and concentration levels for specific substances. Exposure assessment of methylmercury in fish, seafood and mushrooms shows that children not exceeded the recommended tolerable weekly intake. These analyses based on consumption data from VELS with a marginal consumption of tuna. Nevertheless, tuna contributes 5% to methylmercury exposure caused by high levels [34]. New exposure assessment using KiESEL data and the additional information from FPQ should be considered.

Consumption frequencies of tea might be interesting for several reasons. In our point of view, we were interested in each tea species to get better data for exposure assessment to pyrrolizidine alkaloids (PA) [12]. In children, herbal infusion and rooibos were main contributor of PA exposure [35]. In KiESEL, the consumption frequencies of all tea species are more rarely instead of a frequent consumption. Only infants drink baby fennel tea frequently (24%) as often as rarely (27%). In comparison with EsKiMo II, consumption of tea in general is not popular in older children as well. In the total quantity of non-alcoholic beverages, tea has a proportion of 4% in girls and 6% in boys (6–11 years) and 8% in girls and 4% in boys (12–17 years) [36].

EFSA started in the last years a systematic re-evaluation of authorized food additives and therefore also for sweeteners [37]. Calorie-reduced soft drinks contain sweeteners for e.g. aspartame or sucralose. In KiESEL, consumption frequencies increases with the age of children (infants: 0.2%, toddlers 8%, children: 21% consumer). The percentages of consumers of calorie-reduced soft drinks are equal for all socio-economic statuses but the frequency matters. For calorie-reduced soft drinks, the percentages of frequent consumers from families with lower SES were always higher than in the other two groups. Families with lower SES seem to drink calorie-reduced soft drinks more frequently. Under the German National Reduction and Innovation Strategy for Sugar, Fats and Salt in Finished Products, it could be assumed that sugar in soft drinks could be replaced by sweeteners, so that it is of interest whether there is an increased consumption of sweeteners to monitoring the reduction but also from a food safety perspective [38]. Among boys (11%) and girls (13%) from 6–11 years in EsKiMoII-study, lemonade (with or without sweeteners) are the third most frequently consumed beverages [36].

*Rocket salad* is known for possibly containing high contents of nitrate [39]. Children do not seem to be big consumers of rocket salad. Comparing our results with those of a consumer survey asking for seldom eaten foods conducted by BfR 10 years ago, we could see that only 3% of the KiESEL participants are frequent consumers of rocket salad (and 29% additional rare consumers) whereas 12% of the adults of the former consumer survey seemed to be frequent consumers and 57% rare consumers [40]. The small percentages might also result from the fact that rocket salad is no popular food for small children because of its spicy taste.

The consumption of *dark chocolate* was already an issue at the BfR because of the cadmium concentration in cocoa [41]. Dark chocolate consumption is higher for children than for infants or toddlers even though there are much more seldom consumers than frequent consumers. This was similar to the findings of this previous survey for adults. Nevertheless, the consumer percentages for adults were higher than for children. It seems that children prefer milk chocolate to dark chocolate.

## Conclusions

The KiESEL study collected up-to-date information on food consumption for more than 1000 children living in Germany, aged six month up to including five years. These data renew the existing consumption data for this age group collected in 2001/2002 and fill the before existing data gap for the five year old children.

The data collection method is in compliance with EFSA recommendations and some methodological improvements were done in the data collection. The KiESEL study is the first study in Germany which record basic consumption data at home *and* in day care facilities. We support this approach in this age group to complete the overall daily consumption and to enable calculation of the total daily exposure. Further, the KiESEL survey has included a FPQ for rarely consumed foods that are often underrepresented in short-term dietary protocols. The findings from specific foods, e.g., highly contaminated fish species, support this method because of its relevance to risk assessment. Hence, KiESEL is building a comprehensive database for exposure and intake estimates. The gathered information in KiESEL will be used for estimating intake of an assessing risk connected to contaminants, food additives, pesticides or other potentially harmful substances in food. Based on these estimates, food safety evaluations will be done and maximum limits for particular substances in food can be defined.

Additionally, the data of the KiESEL study will be used for estimating energy and nutrient intake to describe the nutritional status of infants, toddlers and children in Germany and to create a scientific basis for policy-makers. Using the data on comparative exposure estimates within the European Union is also possible. The comparable methodology allows trend analyses between the VELS study and the KiESEL study. As all this information is not only of national but also of international interest for exposure measurements and risk assessment, data will be provided to national and international partners such as the World Health Organization.

## Supplementary Information

**Additional file 1. **Supplementary File 

## Data Availability

The datasets generated and analysed during the current study are not yet publicly available. The datasets used and/or analysed during the current study are available from the corresponding author on reasonable request.

## Abbreviations

BfR: German Federal Institute for Risk Assessment
CASMIN: Comparative Analysis of Social Mobility in Industrial Nations
DONALD: DOrtmund Nutritional and Anthropometric Longitudinally Designed Study
EFSA: European Food Safety Authority
EsKiMo: Eating Study as a KiGGS module
FFQ: Food Frequency Questionnaire
FPQ: Food Propensity Questionnaire
FKE: Research institute for child nutrition
GRETA: German Representative Study of Toddler Alimentation
KiESEL: The Children’s Nutrition Survey to Record Food Consumption
KiGGS: German Health Interview and Examination Survey for Children and Adolescents
MRI: Max Rubner-Institute
RKI: Robert Koch Institute
SES: Socio-economic status
VELS: Consumption study to determine the food intake of infants and young children to assess an acute toxicity risk from pesticide residues
